# Predictive value of serological indices for guiding bundle of care to prevent the occurrence of poststroke fatigue for ischemic stroke survivors

**DOI:** 10.1097/MD.0000000000039991

**Published:** 2024-10-04

**Authors:** Qiuping Ma, Jinpan Yang, Lorna Kwai Ping Suen, Jialin Zhang, Chunxiao Yang, Mingyang Zhong

**Affiliations:** a Guangxi University of Chinese Medicine, Nanning, Guangxi, China; b Henan Vocational University of Science and Technology, Zhoukou, Henan, China; c Tung Wah College, Hong Kong, China; d Siping City Central People’s Hospital, Jilin, China.

**Keywords:** bundle of care, ischemic stroke, poststroke fatigue, serological indices

## Abstract

Almost half of ischemic stroke (IS) survivors have poststroke fatigue (PSF) during rehabilitation, which can reduce their rehabilitation compliance and quality of life. The primary link of PSF management is early identification, which can guide bundle of care for prevention. This study aimed to explore the predictive value of serological indicators for guiding bundle of care to prevent the occurrence of PSF among IS survivors. This study was a prospective observational study. A total of 350 patients with IS who were hospitalized in 2 tertiary hospitals in Nanning from October 2022 to September 2023 were selected. The general data of patients and serological indicators within 24 hours of admission were collected. Based on the follow-up results, the patients were divided into the PSF group and the NPSF group. Multivariate logistic regression analysis was used to screen the risk factors affecting the occurrence of PSF, and the receiver operating characteristic curve (ROC curve) method was used to analyze the predictive value of this factor. The incidence of acute-phase PSF among elderly patients with IS was 49.26%. The elevated levels of fasting plasma glucose (FPG) (OR = 1.485, 95% CI: 1.145–1.925, *P* = .003), total cholesterol (TC) (OR = 1.394, 95% CI: 1.013–1.917, *P* = .041), C-reactive protein (CRP) (OR = 1.394, 95% CI: 1.013–1.917, *P* = .041), and homocysteine (Hcy) (OR = 1.370, 95% CI: 1.233–1.524, *P* < .001) were risk factors of PSF in elderly patients with acute IS (*P* < .05). FPG (area under the curve = 0.632), TC (area under the curve = 0.621), CRP (area under the curve = 0.889), and Hcy (area under the curve = 0.807) had a good predictive value for acute-phase PSF, and the combination of the 4 indicators could further improve the predictive efficacy (area under the curve = 0.938, sensitivity 86.2%, specificity 90.7%, *P* < .05). The elevated levels of FPG, TC, CRP, and Hcy could predict the risk of PSF, and the combination of the 4 indicators can effectively improve prediction efficiency and provide a reference for guiding the formulation of bundle nursing programs.

## 1. Introduction

Poststroke fatigue (PSF) is a multi-dimensional fatigue syndrome involving the complexity of the body, emotion, cognition, and society.^[1]^ PSF is mostly manifested as pathological fatigue that cannot be alleviated after rest, and it persists.^[2]^ PSF is a common complication in elderly patients with ischemic stroke (IS), and its incidence is between 42% and 53%.^[3]^ PSF mostly begins in the acute-phase of stroke, and the acute-phase is a critical period for the functional recovery of patients. Studies have shown that the duration of PSF can reach more than 18 months,^[4]^ and almost half of adults will complain of fatigue at any stage after stroke.^[3]^ Long-term fatigue can reduce the rehabilitation compliance and quality of life of elderly patients with IS and even increase the risk of death.^[5,6]^ The Stroke Priority Setting Partnership Steering Group in the UK pointed out that fatigue can seriously affect the quality of life even in people with complete physical recovery. Understanding and reducing fatigue during stroke rehabilitation and long-term care research are among the top 4 areas that medical staff should give priority to.^[7]^ However, in clinical practice, fatigue is usually ignored by medical staff because its symptoms are not prominent. Therefore, conducting early risk assessment for PSF, identifying high-risk patients, promoting their early medical treatment, and transforming passive medical treatment into active health management are necessary.

Bundle nursing refers to the collection of a series of evidence-based nursing measures to solve clinical problems, aiming to promote nursing staff to provide high-quality medical and nursing services to patients.^[8]^ Bundle nursing is the transformation from traditional nursing mode to evidence-based nursing mode.^[9]^ Implementing interventions is an important means of transforming knowledge from research to practice, and comprehensive evidence is necessary to guide the selection and use of interventions.^[10]^ However, considering that our intervention may not have a direct effect on clinical outcomes, the effect of combining multiple interventions may be increased. As an active nursing intervention program, the bundle nursing program has a good clinical effect compared with the conventional single nursing program.^[11]^ Rehabilitation programs based on research evidence provide good rehabilitation opportunities to stroke survivors.^[10]^ Current evidence shows that in the prevention of pressure sores and aspiration and in improving patient care for the satisfaction of patients with stroke, joint care is superior to conventional nursing measures.^[12]^ In reducing the incidence of stroke-associated pneumonia, evidence-based care packages can successfully reduce its incidence.^[13]^ Assessing and identifying high-risk patients is the first step in the implementation of bundle intervention programs. Serological examination is a common clinical examination method. As an existing resource, this method has simple operation, objective data, and easy access. Relevant studies have found that serum indexes such as fasting plasma glucose (FPG), total cholesterol (TC), C-reactive protein (CRP), and homocysteine (Hcy) are related to fatigue.^[14–16]^ However, the predictive value of each serum indicator of acute-phase PSF among elderly patients with IS has not been further explored.

In this study, we aimed to investigate the prevalence of acute-phase PSF among elderly patients with IS, to analyze the relationship between clinical serological indicators (FPG, TC, CRP, and Hcy) within 24 hours after stroke and the occurrence of PSF 14 days after stroke, and to explore the predictive value of serological indicators in guiding bundle nursing to prevent PSF in IS survivors. A comprehensive understanding of the predictive value of FPG, TC, CRP, and Hcy for PSF is helpful for medical staff to identify high-risk patients with PSF early and guide nursing staff in developing bundle nursing programs to prevent the occurrence of PSF. Consequently, the effect of PSF on the rehabilitation of neurological function in elderly patients with IS will decrease, and the quality of life of elderly patients will improve to a certain extent, thereby benefiting their families and society to a certain extent.

## 2. Methods

### 2.1. Participants

This study was a prospective observational study. A total of 350 patients with IS confirmed by cranial MRI or CT from October 2022 to September 2023 in the Department of Neurology of 2 of the top 3 hospitals in Nanning were selected as the research objects. Participants who met the following criteria were included: the diagnosis of IS was made by more than 2 neuroscientists and confirmed by brain CT or MRI; the onset of the IS was 7 days or less; the patient was aged 60 years old; the patients had clear consciousness, sufficient cognitive ability, and audiovisual conditions that met the level of clinical evaluation; patients provided an informed consent and volunteered to participate in the study. The exclusion criteria were as follows: the patient had fatigue before stroke; patients with severe dependence on self-care ability, modified Rankin scale score of ≥ 4 points; patients with severe hearing, vision, communication, cognitive impairment, or severe paralysis, who cannot cooperate with the completion of a credible survey and scale assessment; patients with previous anxiety and depression, sleep disorders, immune system diseases, hematopoietic system diseases, tumor history, combined with severe diseases; patients with incomplete medical records or lost to follow-up. This study received ethical approval from the Hospital (No: GXZYA 2022-031-01), and informed consent were obtained from all patients in this study. All participants in the study provided a written informed consent. Our research followed the principles of the 1964 Declaration of Helsinki.

## 3. Independent variables

The general data of patients were collected through the hospital HIS system, such as age, gender, history of stroke, history of hypertension, history of diabetes, history of coronary heart disease, history of hyperlipidemia, and lesion location. The serological indexes of patients within 24 hours of admission were collected, such as FPG, TC, triglyceride, high-density lipoprotein, low-density lipoprotein, fibrinogen, CRP, uric acid, creatinine, and Hcy.

## 4. Fatigue assessment

Another researcher assessed PSF within 14 days of onset, and the fatigue severity scale (FSS) recommended by the American Heart Association Council on Cardiovascular and Stroke Nursing and Stroke Council was used to assess the patient’s fatigue status.^[17]^ The total average score on the FSS scale was 7 points, and the average score of ≥ 4 points indicated fatigue. The higher the score, the more serious the fatigue. Wu et al translated the FSS scale to Chinese, and Cronbach alpha coefficient of the FSS scale after translation was 0.929,^[18]^ which showed good reliability in screening PSF.

Another researcher followed up with the patients within 14 days of onset. Based on the occurrence of PSF, 167 cases were included in the PSF group and 172 cases in the NPSF group.

## 5. Statistical analysis

Statistical analysis was performed using SPSS 29.0. The measurement data that met the normal distribution and homogeneity of variance were expressed as $\bar{\chi }\pm S$, and a *t*-test was used for comparison. Measurement data with skewed distribution were expressed as median *M*(*Q*_25_, *Q*_75_), which cannot satisfy the normal distribution and homogeneity of variance, which were compared by using the Mann–Whitney *U* test. The count data were expressed by the frequency and composition ratio, and the χ^2^ test was used for comparison. Multivariate logistic regression was used to analyze the risk factors of PSF, and the ROC curve was used to analyze the predictive value of serum indexes. *P* < .05 was considered statistically significant.

## 6. Results

### 6.1. Comparison of general data between the 2 groups

**Figure 1.**
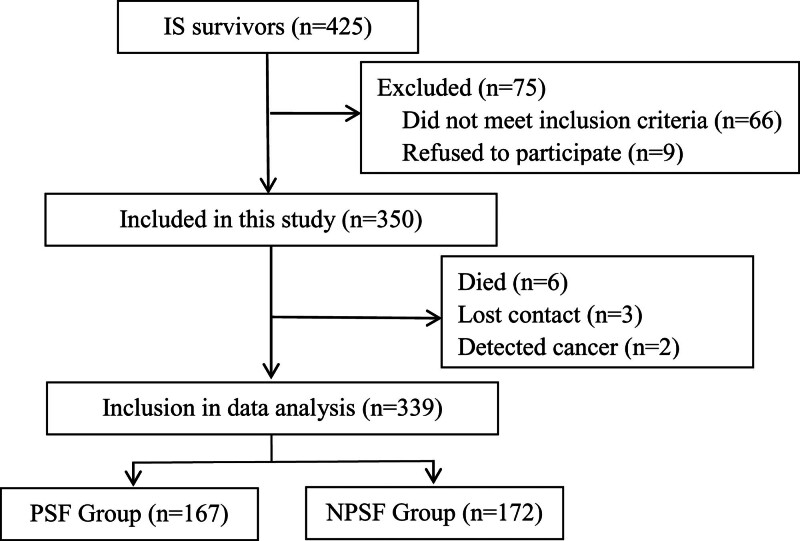
In this study, 425 patients with ischemic stroke (IS) within 7 days of onset were initially included. Seventy-five cases were excluded (66 cases not meeting the inclusion criteria and 9 cases refusing to participate). A total of 350 cases were successfully included as research subjects. During follow-up, 6 cases died, 3 cases were lost to follow-up, and 2 cases withdrew due to cancer. Eventually, 339 patients fully participated in this study. According to whether patients had poststroke fatigue, the 339 patients were divided into 2 groups: the poststroke fatigue group (PSF) (167 cases) and the non-poststroke fatigue group (NPSF) (172 cases).

A total of 425 IS survivors were screened in this study, and 75 cases were excluded. Thus, only 350 patients were included in this study. During the follow-up period, 11 patients were lost to follow-up because of death and other reasons. Finally, we analyzed 339 patients, of which 167 were included in the PSF group and 172 were included in the NPSF group (Fig. 1). The incidence of acute-phase PSF among elderly patients with IS was 49.26%.

The comparison of general data showed that the proportion of previous stroke history, hypertension, diabetes, coronary heart disease, and the mean age level of patients in the PSF group was higher than those in the NPSF group (*P* < .05, Table 1). No significant difference in gender, lesion location, and proportion of hyperlipidemia was found between the 2 groups (*P* > .05, Table 1).

**Table 1:** Comparison of general data between the 2 groups.

Variables	PSF group (n* = *167)	NPSF group (n* = *172)	χ^2^/*t*	*P*-value
Age, mean (SD)	71.54 (9.43)	65.48 (9.23)	5.981	<.001*
Sex, n (%)				
Male	104 (62.28)	109 (63.37)	0.044	.835†
Female	63 (37.73)	63 (36.63)		
Lesion sites, n (%)				
Frontal lobe	63 (37.72)	86 (50.00)	5.226	.073†
Temporal lobe	28 (16.77)	22 (12.79)		
Basal ganglia	76 (45.51)	64 (37.21)		
Previous stroke, n (%)	72 (43.11)	43 (25.00)	12.403	<.001†
Hypertension, n (%)	128 (76.65)	110 (63.95)	6.527	.011†
Diabetes, n (%)	65 (38.92)	41 (23.84)	8.972	.003†
Coronary heart disease, n (%)	23 (13.77)	12 (6.98)	4.226	.040†
Hyperlipidemia, n (%)	51 (30.54)	39 (22.67)	2.687	.101†

NPSF = no poststroke fatigue, PSF = poststroke fatigue.

* By student *t*-test.

† By Chi-square test.

## 7. Comparison of serological indicators between the 2 groups of patients

The results of this study showed that the levels of FPG, TC, Low-density lipoprotein, Fibrinogen, CRP, Creatinine, and Hcy in the PSF group were higher than those in the NPSF group (*P* < .05). No significant difference in Triglyceride, High-density lipoprotein, and Uric acid levels was found between the 2 groups (*P* > .05, Table 2).

**Table 2:** Comparison of the serological indexes between the 2 patient groups.

Variables	PSF group (n = 167)	NPSF group (n* = *172)	*Z/t*	*P*-value
FPG (mmol/L), *M*(*Q*_25_, *Q*_75_)	6.10 (5.40, 7.53)	5.50 (5.10, 6.40)	−4.206	<.001*
TC (mmol/L), mean(SD)	5.04 (1.30)	4.53 (1.07)	4.006	<.001†
TG (mmol/L), *M*(*Q*_25_, *Q*_75_)	1.41 (1.01, 1.98)	1.32 (0.95, 1.78)	−1.630	.103*
HDL (mmol/L), *M*(*Q*_25_, *Q*_75_)	1.15 (0.98, 1.34)	1.18 (1.00, 1.37)	−0.932	.278*
LDL (mmol/L), mean(SD)	3.02 (1.09)	2.64 (0.90)	3.589	<.001†
FIB (g/L), *M*(*Q*_25_, *Q*_75_)	3.59 (3.05, 4.17)	3.22 (2.87, 3.74)	−3.828	<.001*
CRP (mg/L), *M*(*Q*_25_, *Q*_75_)	14.50 (11.20, 16.50)	6.50 (4.30, 7.60)	−12.402	<.001*
UA (μmol/L), mean(SD)	328.37 (98.96)	314.52 (81.44)	1.405	.161†
Cr (μmol/L), *M*(*Q*_25_, *Q*_75_)	80.00 (62.00, 98.00)	70.50 (61.00, 85.00)	−3.039	.002*
Hcy (μmol/L), *M*(*Q*_25_, *Q*_75_)	16.50 (14.10, 18.70)	11.75 (10.13, 14.08)	−9.771	<.001*

Cr = creatinine, CRP = C-reactive protein, FIB = fibrinogen, FPG = fasting plasma glucose, Hcy = homocysteine, HDL = high-density lipoprotein, LDL = low-density lipoprotein, M = median, NPSF = no poststroke fatigue, PSF = poststroke fatigue, Q = percentiles, TC = total cholesterol, TG = triglyceride, UA = uric acid.

* By Mann–Whitney *U* test.

† By Student *t*-test.

## 8. Analysis of risk factors of acute-phase poststroke fatigue among elderly patients with ischemic stroke

Multivariate logistic regression analysis was performed to determine whether the patient had PSF as the dependent variable (no = 0, yes = 1) and identify the indicators with statistically significant differences in univariate analysis as independent variables. The results of multivariate logistic regression analysis showed that the increase of FPG, TC, CRP, and Hcy levels was a risk factor of acute-phase PSF among elderly patients with IS (*P* < .05, Table 3).

**Table 3:** Analysis of risk factors for poststroke fatigue in the acute-phase of elderly IS patients.

Variables	β	SE	Wald χ^2^	*P*-value	OR	95% CI
FPG	0.395	0.132	8.911	.003	1.485	1.145, 1.925
TC	0.332	0.163	4.163	.041	1.394	1.013, 1.917
CRP	0.369	0.045	67.900	<.001	1.446	1.325, 1.579
Hcy	0.315	0.054	33.966	<.001	1.370	1.233, 1.524

95% CI = 95% confidence interval, CRP = C-reactive protein, FPG = fasting plasma glucose, Hcy = homocysteine, OR = odds ratio, SE = standard error, TC = total cholesterol.

### 8.1. Value of fasting plasma glucose, total cholesterol, C-reactive protein, and homocysteine levels in predicting the occurrence of acute-phase PSF among elderly patients with ischemic stroke

**Figure 2.**
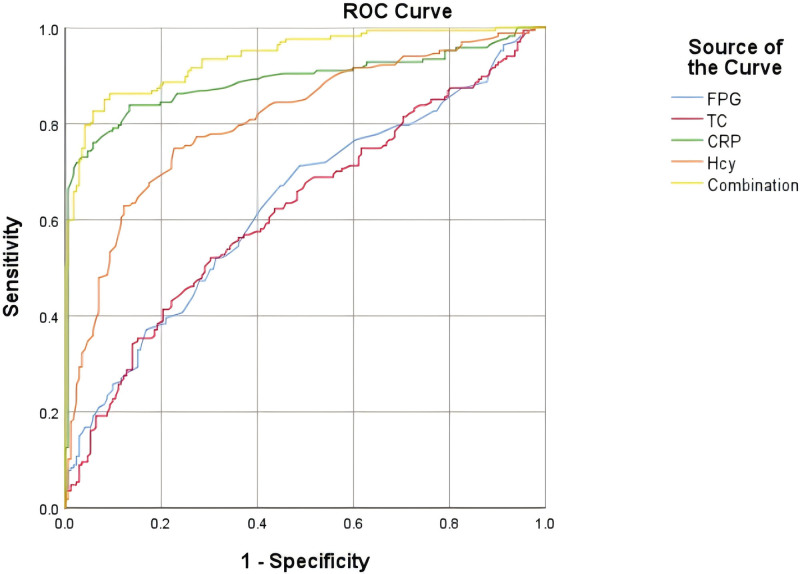
Receiver Operating Characteristic curve (ROC curve) of fasting plasma glucose (FPG), total cholesterol (TC), C-reactive protein (CRP), and Homocysteine (Hcy) levels alone and in combination to predict acute-phase poststroke fatigue among elderly patients with IS. ROC curve analysis was performed with SPSS 26.0 software to obtain the ROC curve graph. The results show that FPG, TC, CRP, and Hcy, used alone or in combination, all have certain predictive value for poststroke fatigue in the acute-phase of elderly patients with IS (area under the curve > 0.5).

ROC curve analysis showed that FPG, TC, CRP, and Hcy alone and in combination had predictive value for acute-phase PSF among elderly patients with IS (AUC > 0.5, *P* < .05, Fig. 2 and Table 4).

**Table 4:** Predictive value of FPG, TC, CRP, and Hcy levels in predicting poststroke fatigue among elderly patients with acute IS.

Variables	AUC	*P*-value	95% CI	Cutoff value	Sensitivity	Specificity	Youden index
FPG	0.632	<.001	0.573, 0.691	5.56 mmol/L	0.713	0.512	0.225
TC	0.621	<.001	0.562, 0.681	5.01 mmol/L	0.521	0.698	0.219
CRP	0.889	<.001	0.851, 0.928	9.35 mg/L	0.838	0.866	0.704
Hcy	0.807	<.001	0.760, 0.854	14.30μmol/L	0.749	0.773	0.522
Combination	0.938	<.001	0.914, 0.963	0.491	0.862	0.907	0.769

95% CI = 95% confidence interval, AUC = area under the curve, CRP = C-reactive protein, FPG = fasting plasma glucose, Hcy = homocysteine, TC = total cholesterol.

## 9. Discussion

PSF is a common complication in elderly patients with acute IS. With the increase of age, the elderly become susceptible to neuroendocrine disorders and persistent low hormone levels. In addition, they can show lower exercise endurance and aggravate their fatigue because of the decrease in skeletal muscle content, strength, and function, which seriously affects the recovery of neurological function and quality of life of patients, as well as increases the mortality rate.^[19]^ This study showed that the incidence of acute-phase PSF in elderly patients with IS was 49.26%, which was consistent with the results of Paudel et al (49.5%).^[20]^ At present, the pathogenesis of PSF remains unclear. A scientific statement issued by the American Heart Association for healthcare professionals indicates that as of now, there is no specific drug for the treatment of PSF.^[17]^ The efficacy of related neuroexcitatory drugs, antidepressants, and traditional Chinese medicines in ameliorating PSF symptoms has not been fully substantiated. Moreover, the adverse reactions and side effects of these medications remain undefined. PSF is a multi-factor syndrome. Previous studies have shown that its risk factors include uncontrollable factors such as gender, advanced age, and lesion site, and controllable factors include coronary heart disease, abnormal inflammatory indicators, and abnormal glucose and lipid metabolism.^[21,22]^ Controlling a single risk factor has limited effect on improving the prognosis of patients with IS, but early prevention from controllable risk factors in accordance with the pathogenesis is an effective way. The Canadian Stroke Best Practice Recommendations: Mood, Cognition, and fatigue following Stroke emphasizes that healthcare professionals should predict the risk of PSF in patients with stroke at an early stage and improve the self-management awareness and competence of high-risk patients promptly by evaluating, educating, and intervening in the whole process of stroke rehabilitation to help high-risk patients alleviate their fatigue symptoms.^[23]^

PSF is a multi-factor phenomenon. The occurrence and development of this symptom are related to multiple factors. For scientific research serving clinical practice and improving the work efficiency of clinical nurses, this study mainly focuses on indicators that are easily accessible clinically and are more objective and reliable. Undoubtedly, psychological factors are of great significance. However, compared with serological factors, psychological factors may be more arduous to quantify or measure. The assessment of psychological state usually relies on self-reporting, interviewing, or psychological testing. The reliability and validity of these methods may be affected by multiple factors such as respondents’ subjectivity, cultural background, and language barriers. Therefore, psychological factors are excluded from this study. In the future, our research group will continue to explore the important value of psychological factors. Regarding preexisting illness, this study included factors such as previous stroke history, hypertension, diabetes, and coronary heart disease. Univariate analysis showed that the above factors were statistically significant (Table 1). However, when further multivariate logistic regression analysis was conducted, the above factors no longer had statistical significance (*P* > .05). Possible reasons are as follows. Insufficient sample size leads to a reduction in the power of statistical tests. In multivariate logistic regression analysis, as the degrees of freedom increase, the critical value of the test also changes. This may cause significant correlations in univariate analysis to become insignificant in multivariate analysis. Certainly, the reason may also be collinearity among independent variables. This may lead to the effects of some independent variables being potentially overestimated or underestimated, thereby covering up some real associations and resulting in insignificant results. Previous studies have pointed out that in multivariate logistic regression analysis, arterial hypertension, atrial fibrillation, ischemic heart disease, and diabetes have no significant correlation with the risk of PSF at any time point after stroke.^[24]^ Therefore, this study focuses on exploring the predictive value of serological factors for PSF. Through multivariate logistic regression analysis, this study found that elevated FPG, TC, CRP, and Hcy levels were risk factors of acute-phase PSF among elderly patients with IS. ROC curve analysis showed that FPG, TC, CRP, and Hcy had predictive values for PSF. The AUC (0.938) of the combined prediction of the 4 indexes was greater than that of the single index. The sensitivity (86.2%), specificity (90.7%), and Youden index (76.9%) were good, and the predictive value of PSF was high.

Elderly patients with acute IS are susceptible to glucose and lipid metabolism disorders. Studies have shown that about 20% to 50% of patients with acute-phase stroke can have glucose and lipid metabolism disorders, such as elevated blood glucose and blood lipid levels.^[25]^ FPG and TC can reflect the body’s glucose metabolism and lipid metabolism function, respectively, which are clinical indicators to evaluate the glucose and lipid metabolism function of patients, and such indicators are related to fatigue.^[16,26]^ Previous studies have found that blood glucose levels and TC in patients with PSF were significantly higher than those in patients with NPSF.^[26,27]^ Meta-analysis confirmed that elevated blood glucose is a risk factor of PSF in patients with acute stroke,^[21]^ which is consistent with the results of this study. Cheng et al^[28]^ confirmed that TC is an independent risk factor of PSF in patients with IS, which is consistent with the results of this study. The increase in FPG level exceeds the threshold value, which can cause mitochondrial damage and promote the lack of energy in the body. In addition, hyperglycemia can disturb the balance of blood fibrinolysis, thereby producing free radicals and lactic acid, damaging the blood–brain barrier, and aggravating the degree of fatigue among elderly patients with IS.^[29]^ The increase in TC level leads to the deceleration of blood flow and the acceleration of lipid deposition, which leads to the stenosis of the vascular lumen caused by mural thrombus and aggravates the ischemic necrosis of brain tissues to induce fatigue. Further ROC analysis in this study showed that the AUC of FPG and TC in predicting acute-phase PSF among elderly patients with IS was 0.632 and 0.621, respectively, and the optimal critical values were 5.56 and 5.01 mmol/L, respectively. This result indicates that medical staff should dynamically monitor and control FPG and TC levels in the acute-phase and instill self-health management concepts into elderly IS patients with glucose and lipid metabolism disorders to help them enhance their awareness of self-management of blood glucose and blood lipids, learn relevant knowledge and skills, form self-management groups, and encourage peer reminders to supervise 1 another, thereby reducing the risk of PSF.

The inflammation hypothesis is considered to be an important mechanism for the occurrence of cardiovascular and cerebrovascular diseases. The abnormal physiological concentration of serum inflammatory factors can induce PSF by disrupting metabolism and interfering with the synthesis and release of neurotransmitters.^[30]^ Relevant studies have shown significant differences in serum CRP and Hcy levels between patients with PSF and those with NPSF.^[14,31]^ Wang et al^[32]^ found that elevated serum CRP and Hcy levels were risk factors of PSF in elderly patients with cerebral infarction, which was consistent with the results of this study. CRP induces a large number of pro-inflammatory factors, affects the integrity of the brain neurotransmitter system and neurons, and interferes with the expression of brain-derived neurotrophic factors to induce fatigue.^[33]^ Hcy may destroy the balance between coagulation and fibrinolysis, increase the content of oxygen free radicals, lead to vascular endothelial peroxidation, and then damage cell energy metabolism to induce PSF.^[34]^ The results of further ROC analysis in this study showed that the AUC of CRP and Hcy in predicting acute-phase PSF among elderly patients with IS was 0.889 and 0.807, respectively. Therefore, serum CRP and Hcy levels in acute-phase stroke and the body’s inflammatory response can be controlled, and elderly patients can be guided to establish healthy lifestyles such as healthy diet, moderate exercise, smoking cessation, and alcohol restriction to alleviate the inflammatory response, thereby reducing the risk of acute-phase PSF.

Early identification of factors that can predict the increased risk of PSF will help medical staff design bundle nursing programs and provide stroke health counseling for patients and their family members. This study suggests that after assessing the risk, medical staff should develop an evidence-based bundle nursing program based on the principles of stable control of glucose, lipid metabolism, and regulation of inflammatory response for patients at high-risk of PSF. However, this study still has some limitations. First, the sample size is small, which may lead to sampling and selection bias, so the sample size must be expanded in the future. Second, serological indicators were not dynamically monitored and were only measured once on admission, but the level of serum indicators fluctuated with time, and dynamic monitoring should be carried out in the future. Third, PSF is a multi-factor phenomenon, which may not be captured by a single result measurement. In this study, the FSS scale was used to evaluate the fatigue status. Although the scale showed good performance, objective indicators to support this result are still lacking. In the future, we can further explore the relationship between dynamic serum indicators and PSF through multi-center, large-sample longitudinal studies and try to transform the predicted research results into practical tools in the tertiary prevention process to promote clinical application.

## 10. Conclusion

Elevated serum FPG, TC, CRP, and Hcy levels are risk factors of acute-phase PSF among elderly patients with IS. Serum FPG, TC, CRP, and Hcy have good early predictive value for PSF. The combined use of the 4 indicators can effectively improve prediction efficiency, help the early identification of PSF, and formulate a bundle nursing plan. A package of intervention methods such as the stable control of blood glucose, blood lipid metabolism, and regulation of inflammatory response in IS survivors may effectively prevent the occurrence of PSF.

## Acknowledgments

The authors thank the patients who participated in this study and the staff of the department of Neurology.

## Author contributions

**Conceptualization:** Qiuping Ma.

**Data curation:** Jinpan Yang, Jialin Zhang, Chunxiao Yang, Mingyang Zhong.

**Formal analysis:** Jinpan Yang, Lorna Kwai Ping Suen.

**Funding acquisition:** Qiuping Ma.

**Methodology:** Jinpan Yang.

**Project administration:** Qiuping Ma.

**Resources:** Jinpan Yang, Jialin Zhang, Chunxiao Yang, Mingyang Zhong.

**Supervision:** Lorna Kwai Ping Suen.

**Writing – original draft:** Qiuping Ma, Jinpan Yang, Lorna Kwai Ping Suen.

**Writing – review & editing:** Qiuping Ma, Jinpan Yang.
